# Synthesis and Biological Evaluation of Aminonaphthols Incorporated Indole Derivatives

**DOI:** 10.1155/2014/673206

**Published:** 2014-09-10

**Authors:** Saundane Anand Raghunath, Kirankumar Nandibeoor Mathada

**Affiliations:** Department of Post-Graduate Studies and Research in Chemistry, Gulbarga University, Gulbarga, Karnataka 585 106, India

## Abstract

An efficient one pot condensation of naphthols (1), 2,5-disubstituted indole-3-carboxaldehydes (2), and secondary amines (3) has been achieved using dichloromethane as a solvent, stirring at room temperature. Some of the new [(disubstituted amino)(5-substituted 2-phenyl-1*H*-indol-3-yl)methyl]naphthalene-ols (4) derivatives were prepared in good yields. The significant features of this method are simple work-up procedure, inexpensive nontoxic solvent, shorter reaction times, and excellent product yields. The structures of newly synthesized compounds (**4a–r**) are confirmed by their elemental analysis, FTIR, ^1^H and ^13^C NMR, and mass spectral data. These compounds were screened for their *in vitro* antioxidant, antimicrobial, antitubercular, and anticancer activities. Among the synthesized compounds (**4a–r**), the compound **4e** exhibited highest activity for radical scavenging and ferric ions reducing antioxidant power activities; compounds **4b**, **4h**, and **4k** showed good metal chelating activity. Compounds **4n** and **4q** showed excellent antimicrobial activities with MIC value 08 *µ*g/mL against tested strains. Compounds **4h**, **4k**, **4n**, and **4q** exhibited promising antitubercular activity with MIC value 12.5 *µ*g/mL. Compounds **4k** and **4q** exhibited 100% cell lysis at concentration 10 *µ*g/mL against MDA-MB-231 (human adenocarcinoma mammary gland) cell lines.

## 1. Introduction

The search and evaluation of chemical compounds and their derivatives with a specific pharmacological activity are a demanding task in the drug discovery process. During last few years, the synthesis of aminoalkylnaphthols has received special attention from the scientific community because of their significant biological [1] and catalytic [2] properties. Multicomponent reactions (MCRs) have emerged as an efficient and powerful tool in modern organic chemistry for the synthesis of biologically potent molecules from readily available substrates without isolation of intermediates in minimal time with maximum selectivity. MCRs have proved to be very powerful and efficient bond-forming tool in synthetic chemistry; the reactions are flexible, of high atom economy, and of high purity with excellent yields [3].

Oxidative stress is a well-known mechanism that is responsible for the development of vascular damage. Oxidative stress results from an imbalance between (i) an overproduction of reactive oxygen species (ROS) by the different cellular sources such as the mitochondrial respiratory chain, nicotinamide adenine dinucleotide phosphate hydride oxidases (NADPHOXs or NOX), xanthine oxidase, lipoxygenases, cytochrome-P450, and other oxidases and (ii) decreased cellular and plasma antioxidant defenses [4]. Antioxidants inhibit the generation of ROS and the subsequent formation of lipid peroxidation products, thereby preventing both oxidative and carbonyl stress. Most antioxidants prevent down low-density lipoproteins (LDL) oxidation in cell-free systems and delay the formation of atherosclerotic lesions in animal models for atherosclerosis such as apo E-/-mice [5]. Discrepancies exist about their efficiency against atherosclerosis in humans, this resulting at least in part from the bioavailability within the plaque, their chemical nature, and inability to scavenge reactive carbonyl compounds (RCCs) once adducts are formed on proteins [6]. However, antioxidants remain very efficient in preventing the early atherosclerotic lesions and inflammatory events implicated in the evolution of lesions towards more advanced states [5, 6].

There are some life-threatening diseases; among them tuberculosis (TB) is one of the major life-threatening chronic infections. TB is a specific communicable disease caused by* Mycobacterium tuberculosis (Mtb)*. It affects both the pulmonary and the nonpulmonary tissues. These bacilli are microscopic and were first discovered in 1882. This disease is general or local, acute or chronic. According to WHO report, around one-third of the world's population is infected with TB, resulting in 2-3 million people are going to die annually [7]. The individuals with HIV-positive patients are highly susceptible to* Mtb* with 50-fold risk increases over HIV-negative patients [8]. Every year, about 0.35 million people living with HIV die from TB. Another major problem in TB therapy is the fact that* Mtb* became more resistant to the antituberculosis (anti-TB) drugs [9]. Furthermore, in recent times, the appearance of multidrug-resistant TB (MDR-TB) occurs, which is a form of TB that does not respond to the first- and second-line drugs [10]. TB drugs have become a serious task to TB control and its treatment. Hence, there is an increased demand to develop new tuberculosis agents effective against pathogens resistant to current treatment.

Among women worldwide, breast cancer is the most common cause of cancer death. The latest statistic indicated that about 600,000 women die from the breast cancer disease annually worldwide [11]. MDA-MB 231 cells have high invasive ability that can contribute to metastasis [12, 13]. Most chemotherapeutic drugs induce apoptosis in cancer cell [14]. Apoptosis is considered as a significant form of cancer cell death after treatment with cytotoxic drugs and has been recognized as a standard strategy for the selection of anticancer drug [15, 16]. Thus, there is a great need for new alternative agents for the prevention and treatment of breast cancer.

The indole framework is a medicinally relevant scaffold and has been widely identified as a privileged structure of pharmacophore [17]. Indole scaffold is present in thousands of isolated natural products and also synthetic compounds constitute an important class of therapeutic agents in medicinal chemistry such as antimicrobial [18], antioxidant [19], antiviral [20], anti-HIV, antimalarial [21], and antituberculosis products [22]. Naturally occurring indole derivatives melatonin (I), serotonin (II), tryptophan (III), and indole-3-propionic acid (IV) influence many important biochemical processes, for example, acting as an antioxidant and playing an important role in the immune system [23–28].

In view of Figure 10 and in continuation of our research on the synthesis of biologically active molecules [29–32], in present investigation, we report the synthesis, antioxidant, antimicrobial, antitubercular, and anticancer activities of novel indole derivatives.

## 2. Result and Discussion

### 2.1. Chemistry

**Scheme 1:**
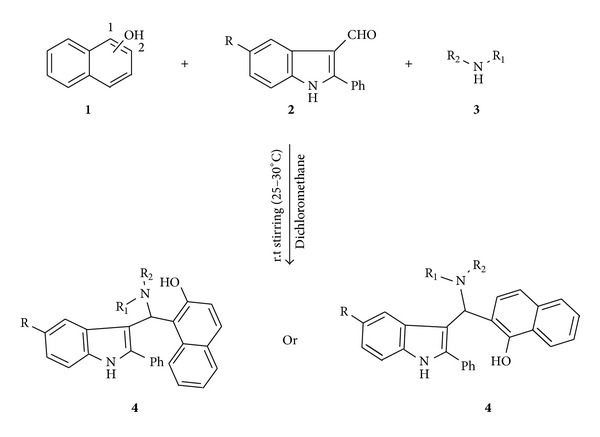
One pot synthesis of indole derivatives.

The one pot synthesis of titled compounds 1-[(5-substituted-2-phenyl-1*H*-indol-3-yl)(piperidin-1-yl)methyl]naphthalene-2-ol and 1-[(dimethylamino)(5-substituted-2-phenyl-1*H*-indol-3-yl)methyl]naphthalene-2-ol and their -1-ol isomers was achieved by condensation of 1- or 2-naphthol and secondary amines with 2,5-disubstituted indole-3-carboxaldehydes in dichloromethane stirring at room temperature (Scheme 1 and Table 1). The progress of the reaction was monitored by TLC. After the completion of the reaction as checked by TLC, dichloromethane was pumped out by rotary evaporation. The crude product was purified directly by crystallization from ethanol.

The structures of all newly synthesized compounds (**4a–r**) were confirmed from their elemental analysis, IR, ^1^H NMR, ^13^C NMR, and mass spectral data. Compound** 4b **in its IR spectrum exhibited characteristic absorption bands at 3126 and 3049 cm^−1^ due to –OH and indole-NH functions, respectively. In its ^1^H NMR spectrum, the downfield signal appeared at 12.60 ppm as singlet was assigned to –OH (confirmed by deuterium exchange) signal at 9.90 ppm, as singlet attributed to the indole-NH, whereas the fourteen aromatic protons resonated as multiplet between 7.30 and 7.82 ppm. The singlet at 5.01 ppm was assigned to single proton of N–C–H, signal at 3.37 ppm appeared due to four protons of two chemically equivalent –CH_2_ groups of piperidine ring, signal at 2.55 ppm was assigned to triplet due to two protons of –CH_2_ group of piperidine ring, and one more signal at 1.11 ppm exhibited due to multiplet four protons of chemically equivalent two CH_2_ groups of piperidine ring. In its ^13^C NMR spectrum, the signal appeared at 150.44 ppm due to C–OH, whereas carbon C–Cl resonated at 128.51 ppm. Further the structure of** 4b **is confirmed by its isotopic molecular ion peak which is in agreement with molecular weight and nitrogen rule.

### 2.2. Antioxidant Activity

#### 2.2.1. 1-Diphenyl-2-picrylhydrazyl (DPPH) Radical Scavenging Activity (RSA)

The synthesized compounds (**4a–r**) were screened for their free radical scavenging activity using DPPH method [33]. This model of radical scavenging activity by DPPH radical is extensively applied to evaluate the antioxidant activity in shorter time. The odd electron in the DPPH free radical gives a strong absorption band at 517 nm, which is purple in color. This property makes it suitable for spectrometric studies. The DPPH assay has often been used to estimate the antiradical activity of antioxidant. The free radical scavenging capacities of the synthesized compounds were measured at different concentrations (25, 50, 75, and 100 *μ*g/mL in methanol) in presence of freshly prepared solution of stable free radical DPPH in methanol. The synthesized compounds react with DPPH radical and convert it into 1,1-diphenyl-2-picrylhydrazine. The extent of decolorization is an indicative potentiality of antioxidant behavior of a particular compound. Butylated hydroxyanisole (BHA), tertiary butylated hydroquinone (TBHQ), and ascorbic acid (AA) are used as reference standards.

**Figure 1:**
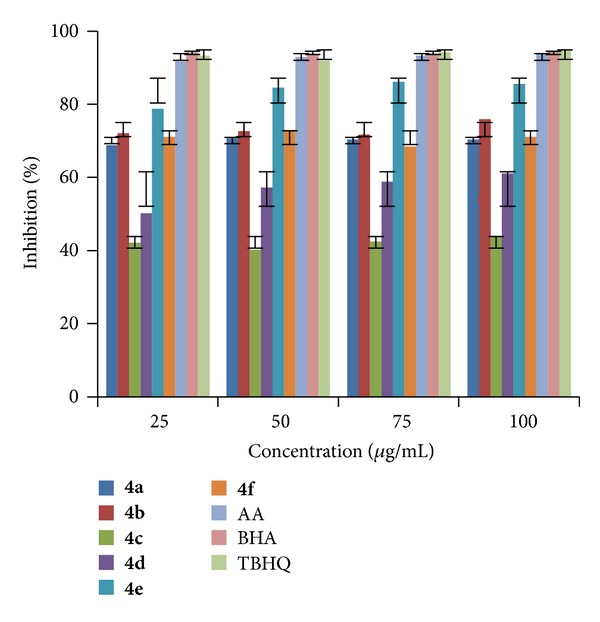
RSA of compounds (**4a–f**).

**Figure 2:**
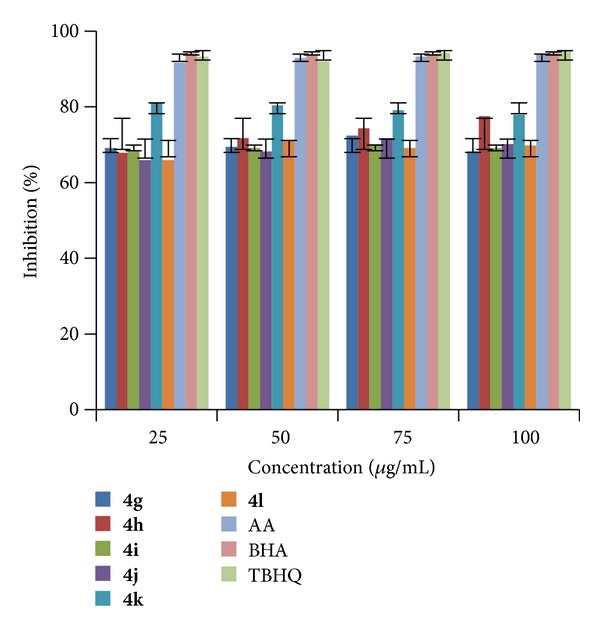
RSA of compounds (**4g–l**).

**Figure 3:**
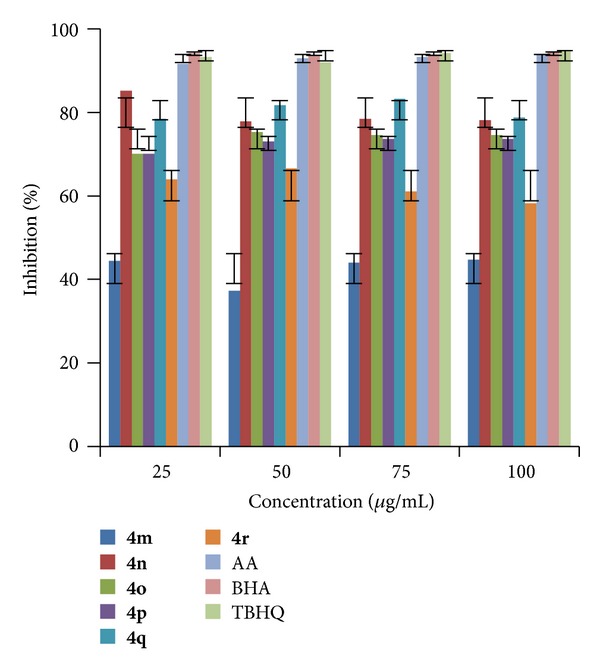
RSA of compounds (**4m–r**).

The analysis of results indicated that (Figures 1, 2, and 3) compound** 4e **exhibited highest radical scavenging activity 86.17% at a concentration of 75 *μ*g/mL. Compounds** 4n**,** 4q, **and** 4r** showed excellent radical scavenging ability of 85.20, 83.27, and 83.25% at concentrations 25, 75, and 75 *μ*g/mL, respectively, and the rest of the compounds showed moderate activity when compared to standards. It is found that IC_50_ values (Table 2) of all the compounds are <25 which is equivalent to all the three tested standards.

#### 2.2.2. Ferric Ions (Fe^3+^) Reducing Antioxidant Power (FRAP)

The ferric ion (Fe^3+^) is a relatively biologically inactive form of iron. However, it can be reduced to the active ferrous ion (Fe^2+^) depending on condition particularly pH [34] and oxidizing back through Fenton type reaction with the production of hydroxyl radical or Haber-Weiss reaction with superoxide anions. Reducing power is to measure the reductive ability of an antioxidant and it is evaluated by the transformation of Fe^3+^ to Fe^2+^ by donation of an electron in the presence of test compounds. Therefore, the Fe^2+^ can be monitored by measuring the formation of Perl's Prussian blue at 700 nm.

**Figure 4:**
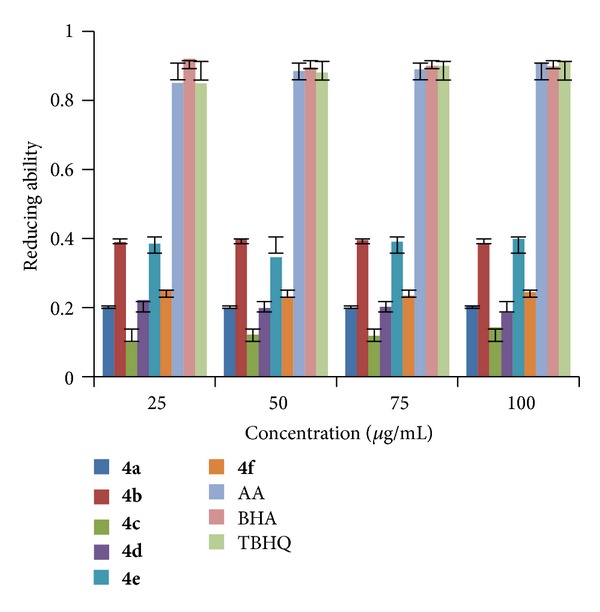
FRAP of compounds (**4a–f**).

**Figure 5:**
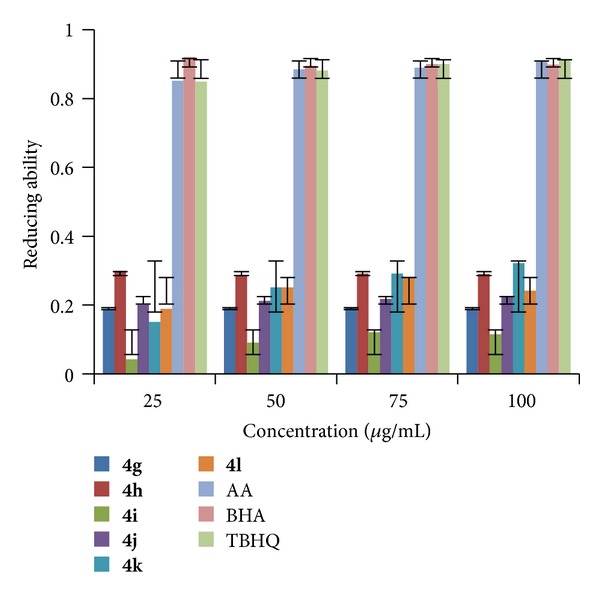
FRAP of compounds (**4g–l**).

**Figure 6:**
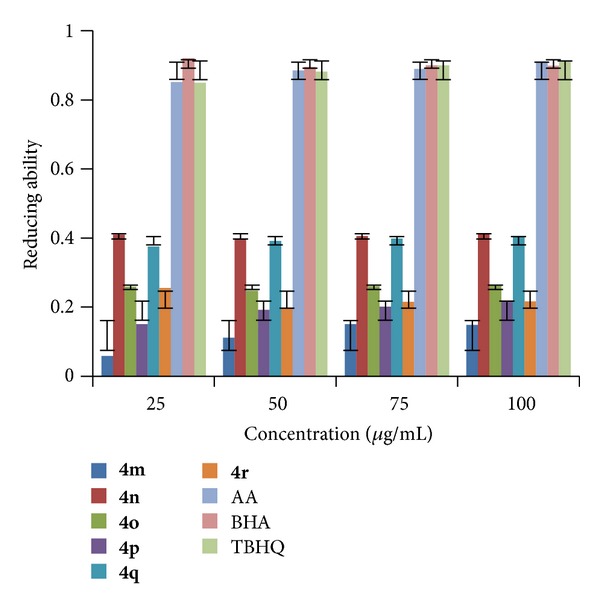
FRAP of compounds (**4m–r**).

The FRAP of synthesized compounds (**4a–r**) was determined at different concentrations (25, 50, 75, and 100 *μ*g/mL in methanol) at pH 6.6 using literature method [35]. The increase in absorbance at 700 nm indicates the increase in reducing ability of a compound. The results shown in Figures 4, 5, and 6 indicated that the compounds** 4e**,** 4n,** and** 4q** exhibited good reducing power activity at concentration 100 *μ*g/mL.

#### 2.2.3. Ferrous (Fe^2+^) Metal Ion Chelating Activity

Among the transition metals, iron is known as the most important lipid oxidation prooxidant due to its high reactivity. The effective ferrous ions chelators may also afford protection against oxidative damage by removing iron (Fe^2+^) that may otherwise participate in hydroxyl radical generating Fenton type reactions [36]:

(1) $$\begin{array}{c} {\text{Fe}}^{2+}+{\text{H}}_{2}{\text{O}}_{2}⟶{\text{Fe}}^{3+}+{\text{OH}}^{-}+{\text{OH}}^{∙} \end{array}$$

Ferric (Fe^3+^) ions also produce radicals from peroxides although the rate is tenfold less than that of ferrous (Fe^2+^) ions [37]. Ferrous ion is the prooxidant among the various species of metal ions [38]. Minimizing ferrous (Fe^2+^) ion may afford protection against oxidative damage by inhibiting production of reactive oxygen species (ROS) and lipid production. Ferrozine can quantitatively form complex with ferrous ions in this method. In the presence of chelating agents, the complex formation is disrupted resulting in a decrease in red color of the complex. Measurement of color reduction therefore allows estimating the metal chelating activity of the coexisting chelators. Lower absorbance indicates higher metal chelating activity.

**Figure 7:**
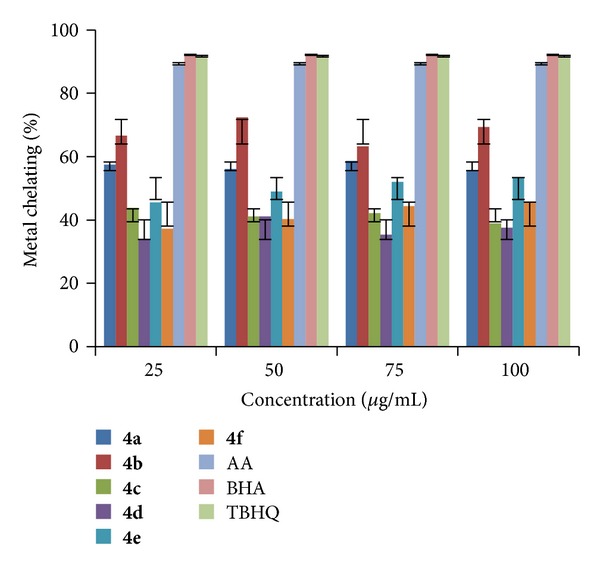
Metal chelating activity of compounds (**4a–f**).

**Figure 8:**
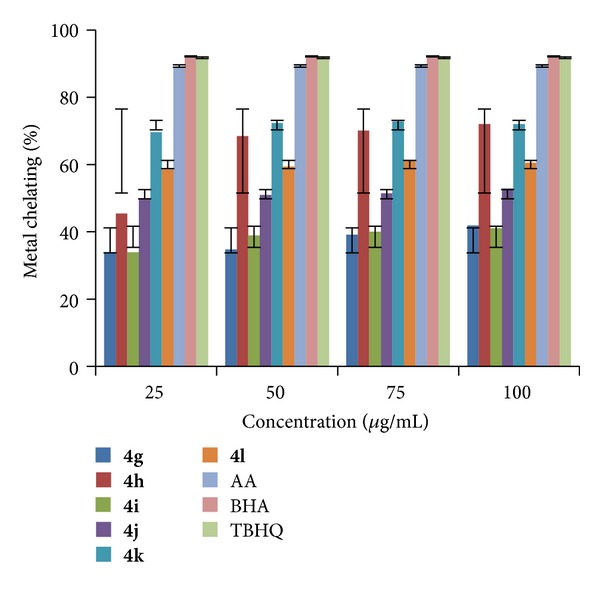
Metal chelating activity of compounds (**4g–l**).

**Figure 9:**
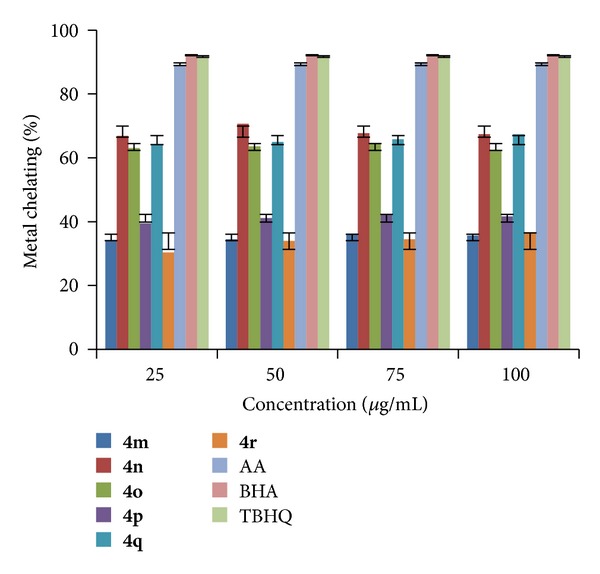
Metal chelating activity of compounds (**4m–r**).

**Figure 10:**
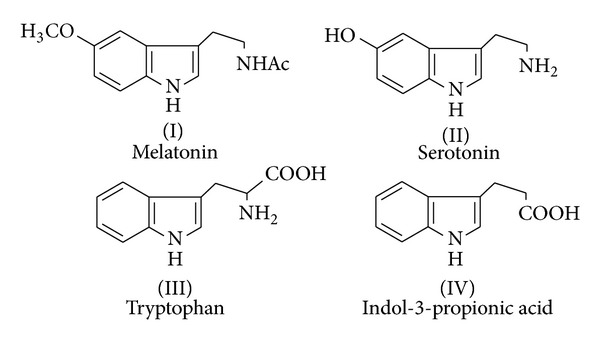
Indole derivatives with antioxidant activity.

All the newly synthesized indole derivatives (**4a–r**) were screened for their metal chelating activity at concentrations 25, 50, 75, and 100 *μ*g/mL in methanol using reported method [39]. The results were compared with the results obtained for standards BHA, TBHQ, and AA. The results shown in Figures 7, 8, and 9 indicated that the compounds** 4b, 4h,** and** 4k** exhibited good metal chelating activity of 72.33, 72.05, and 72.87% at 50, 100, and 75 *μ*g/mL, respectively, whereas other compounds exhibited either moderate or poor chelating activity. These results suggested that the compounds which exhibited good chelating activity interfered with the formation of ferrous and ferrozine complex.

### 2.3. Antimicrobial Activity

All the newly synthesized compounds (**4a–r**) were assessed for their* in vitro* antibacterial activity against four representative bacterial species, namely,* Escherichia coli *(MTCC-723),* Staphylococcus aureus *(ATCC-29513),* Klebsiella pneumonia *(NCTC-13368), and* Pseudomonas aeruginosa (MTCC-1688) *using gentamycin as reference. Determination of MIC was done using the serial dilution method [40, 41]. The materials used were 96-well plates, suspension of microorganism (0.5 McFarland), Muller-Hinton broth (Himedia), and stock solutions of each substance to be tested (2048 *μ*g/mL in DMSO). The following concentrations of the substances to be tested were obtained in the 96-well plates: 1024, 512, 128, 64, 32, 16, 8, 4, and 2 *μ*g/mL. After incubation at 37°C for 18–24 h, the MIC for each tested substance was determined by Bio-Rad Elisa reader (microplate reader S/N 12883). The results are tabulated in Table 3.

*In vitro* antifungal activity of the synthesized compounds (**4a–r**) was assessed against four representative fungal species, namely,* Aspergillus oryzae *(MTCC-3567^T^),* Aspergillus niger *(MTCC-281),* Aspergillus flavus *(MTCC-1973), and* Aspergillus terreus *(MTCC-1782) using fluconazole as a reference, by serial dilution method [42, 43].

The minimal inhibitory concentration (MIC) values are obtained by the broth microdilution method and were tabulated in Table 4. Synthesized compounds have comparable and similar inhibitory effects (low to moderate MIC values 08 and 512 *μ*g/mL). The antibacterial activity results revealed that compound** 4n** showed excellent activity with MIC 08 *μ*g/mL against* K. pneumonia* and MIC 16 *μ*g/mL against* E. coli*,* S. aureus, and P. aeruginosa. *Compound** 4q** showed good activity with MIC 08 *μ*g/mL against* S. aureus* and* P. aeruginosa *andMIC 16 *μ*g/mL against* E. coli *and* K. pneumonia*. Compounds** 4h** and** 4k** exhibited good activity with MIC 16 *μ*g/mL against* E. coli *and* S. aureus.*

On the other hand, the antifungal activity results revealed that compound** 4n **exhibited excellent activity with MIC 08 *μ*g/mL against* A. oryzae, A. niger,* and* A. terreus *and MIC 16 *μ*g/mL against* A. flavus* and compound** 4q **exhibited MIC 08, 16, and 16 *μ*g/mL against* A. niger*,* A. flavus,* and* A. terreus,* respectively. The rest of the compounds showed moderate activity against all the tested fungi.

### 2.4. Antitubercular Activity

The antitubercular activity of compounds (**4a–r**) was assessed against* M. tuberculosis* (ATTC-27294) using the microplate almar blue dye assay (MABA) [44]. The final drug concentrations tested were 100 to 0.2 *μ*g/mL compared with standards pyrazinamide 3.125 *μ*g/mL and streptomycin 6.25 *μ*g/mL. The MIC was defined as the lowest drug concentration which prevented a color change from blue to pink. The results are shown in Table 4.

Compounds** 4h**,** 4k**,** 4n,** and** 4q** exhibited promising activity with MIC 12.5 *μ*g/mL. And the rest of the compounds exhibited moderate activity with MIC 25 and 50 *μ*g/mL.

### 2.5. Anticancer Activity

*MTT Solution Preparation.* 10 mg MTT in 10 mL of Hanks balanced solution was prepared.

*Cell Culture.* The cells were maintained in 96-well microtiter plate containing MEM media supplemented with 10% heat inactivated fetal calf serum (FCS), containing 5% of mixture of gentamycin, penicillin (100 units/mL), and streptomycin (100 *μ*g/mL) in the presence of 5% CO_2_ at 37°C for 3-4 days. After 3-4 days, the supernatant was removed, and MEM media were replaced with Hanks balanced solution supplemented with gentamycin, penicillin, and streptomycin and incubated overnight.

*Cytotoxicity Assay*.* In vitro* growth effect of test compound was assessed by calorimetric method [45]. Determination of conversion of MTT into “Formazon blue” by living cells was done. The supernatant was removed from the plate, and then fresh Hanks balanced salt solution was added and treated with different concentrations of compounds diluted with DMSO. Control group contains only DMSO. After 24 h incubation at 37°C in a humidified atmosphere of 5% CO_2,_ the medium was replaced with MTT solution (100 *μ*g/mL, 1 mg/mL in sterile Hanks balanced solution) and kept for 4 h for incubation. The supernatant was carefully aspirated, the precipitated crystals of “Formazon blue” were solubilized by adding DMSO (200 *μ*g/mL), and absorbance was measured at *λ* 570 nm.

The results represent the mean of three readings. The concentration at which the absorbance of treated cells was reduced by 50% with respect to the untreated control was calculated using the following formula:

(2) Surviving  cells(%) =Mean  absorbance  of  test  compoundMean  absorbance  at  control×100.

Based on the antioxidant result, some of the potent compounds were screened for their cytotoxic activity against MDA-MB-231 (human adenocarcinoma mammary gland) cell lines using standard drugs (Table 5).* In vitro* growth effect of test compounds revealed that compounds** 4k** and** 4q** exhibited 100% cell lysis at concentration 10 *μ*g/mL. The compound** 4n** exhibited 100% cell lysis at concentration 20 *μ*g/mL, and the rest of screened compounds showed moderate activity against MDA-MB-231 (human adenocarcinoma mammary gland) cell lines at concentration of 30 *μ*g/mL.

## 3. Experimental

### 3.1. Analysis and Physical Measurements

Elemental analysis was obtained from Perkin Elmer 2400 CHN elemental analyzer which is microprocessor based instrument. All the compounds gave C, H, and N analysis within ±0.4%. IR spectra of the synthesized compounds were recorded as KBr pellets on a Perkin-Elmer Spectrum RX-IFTIR instrument covering the range 4000–400 cm^−1^. The ^1^H NMR and ^13^C NMR spectra were recorded using DMSO-*d*_6_ as a solvent with a BRUKER NMR 500 MHz and 125 MHz spectrometer, respectively. The chemical shift values are expressed in ppm (*δ* scale) using tetramethylsilane as an internal standard. The mass spectral measurements were carried out by electron impact method on JEOL GC mate spectrometer at 70 eV.

### 3.2. Methods

*General Procedures. *Laboratory chemicals were supplied by Merck and Himedia Ltd. and were of high purity grade; solvents were distilled and dried before use. Melting points of the synthesized compounds were determined by electrothermal apparatus using open capillary tubes. The purity of the compounds was checked by TLC using silica gel-G coated aluminium plates (Merck) and spots were visualized by exposing the dry plates to iodine vapors. The precursors 2,5-disubstituted indole-3-carboxaldehydes (**2a–c**) were prepared by the literature method [46].

### 3.3. Preparation Method and Physical Data of Synthesized Compounds **(4a–r)**

2,5-Disubstituted indole-3-carboxaldehydes (0.001 mol), secondary amine (0.0011 mol), 1- or 2-naphthol (0.001 mol), and dichloromethane (5 mL) were introduced in a 50 mL round bottom flask. The resulting mixture was stirred vigorously with a magnetic bar on a magnetic stirrer for 2-3 hours at room temperature (25–30°C). The progress of the reaction was monitored by TLC. After the completion of the reaction as checked by TLC, dichloromethane was pumped out by rotary evaporation. The crude product was purified directly by crystallization from ethanol (Scheme 1) (Table 1).

#### 3.3.1. Preparation of 1-[(2-Phenyl-1*H*-indol-3-yl)(piperidin-1-yl)methyl]naphthalene-2-ol **(4a)**

Yield = 82%. m.p. 186–188°C; Anal. Calcd. (%) for C_30_H_28_N_2_O: (Mol. Wt. = 432) C, 83.30; H, 6.48; N, 6.48. Found (%): C, 83.34; H, 6.52; N, 6.50. FT-IR (KBr, cm^−1^): 3133 br, *ν*(OH); 3099 *ν*(indole-NH). ^1^H NMR (*d*_6_-DMSO, ppm): 12.61 (s, 1H, phenolic OH, exchangeable with D_2_O); 9.94 (s, 1H, indole-NH); 7.31–7.80 (m, 15H, ArH); 4.98 (s, 1H, N–CH); 3.39 (t, 4H, 2x CH_2_); 2.50 (m, 2H, CH_2_); 1.21 (m, 4H, 2x CH_2_).

#### 3.3.2. Preparation of 1-[(5-Chloro-2-phenyl-1*H*-indol-3-yl)(piperidin-1-yl)methyl]naphthalene-2-ol **(4b)**

Yield = 85%; m.p. 258–260°C; Anal. Calcd. (%) for C_30_H_27_ClN_2_O: (Mol. Wt. = 466) C, 77.17; H, 5.78; N, 6.00. Found (%): C, 77.16; H, 5.83; N, 6.02. FT-IR (KBr, cm^−1^): 3126 br, *ν*(OH); 3049 *ν*(indole-NH); 750, *ν*(C–Cl). ^1^H NMR (*d*_6_-DMSO, ppm): 12.60 (s, 1H, phenolic OH, exchangeable with D_2_O); 9.96 (s, 1H, indole-NH); 7.30–7.82 (m, 14H, ArH); 5.01 (s, 1H, N–CH); 3.37 (t, 4H, 2x CH_2_); 2.55 (m, 2H, CH_2_); 1.11 (m, 4H, 2x CH_2_). ^13^C NMR (*d*_6_-DMSO, ppm): 185.99 (C–Cl) 150.45 (C–OH), 134.91, 130.58, 130.33, 129.81, 129.50, 127.47, 127.41, 124.18, 120.58, 114.14, 113.43, 40.49, 40.33, 40.25, 40.16, 39.99, 39.83, 39.66, 39.49. MS (EI) *m*/*z*: (M^+^, M^+2^): 466, 488 (4.1%, 1.1%).

#### 3.3.3. Preparation of 1-[(5-Methyl-2-phenyl-1*H*-indol-3-yl)(piperidin-1-yl)methyl]naphthalene-2-ol **(4c)**

Yield = 79%; m.p. 252–254°C; Anal. Calcd. (%) for C_31_H_30_N_2_O: (Mol. Wt. = 446) C, 83.37; H, 6.77; N, 6.27. Found (%): C, 83.40; H, 6.72; N, 6.28. FT-IR (KBr, cm^−1^): 3118 br, *ν*(OH); 3051 *ν*(indole-NH). ^1^H NMR (*d*_6_-DMSO, ppm): 12.59 (s, 1H, phenolic OH, exchangeable with D_2_O); 9.90 (s, 1H, indole-NH); 7.32–7.89 (m, 14H, ArH); 4.98 (s, 1H, N–CH); 3.35 (t, 4H, 2x CH_2_); 2.45 (m, 2H, CH_2_); 2.33 (s, 3H, C–CH_3_); 1.11 (m, 4H, 2x CH_2_).

#### 3.3.4. Preparation of 2-[(2-Phenyl-1*H*-indol-3-yl)(piperidin-1-yl)methyl]naphthalene-1-ol **(4d)**

Yield = 89%; m.p. 166–168°C; Anal. Calcd. (%) for C_30_H_28_N_2_O: (Mol. Wt. = 432) C, 83.30; H, 6.52; N, 6.48. Found (%): C, 83.35; H, 6.48; N, 6.42. FT-IR (KBr, cm^−1^): 3120 br, *ν*(OH); 3035 *ν*(indole-NH); 2900, *ν*(Ar CH–Str); 2811, *ν*(CH–Str); 1559, *ν*(ter Amine N–C). ^1^H NMR (*d*_6_-DMSO, ppm): 12.59 (s, 1H, phenolic OH, exchangeable with D_2_O); 9.99 (s, 1H, indole-NH); 7.32–7.79 (m, 15H, ArH); 4.99 (s, 1H, N–CH); 3.35 (t, 4H, 2x CH_2_); 2.50 (m, 2H, CH_2_); 1.18 (m, 4H, 2x CH_2_).

#### 3.3.5. Preparation of 2-[(5-Chloro-2-phenyl-1*H*-indol-3-yl)(piperidin-1-yl)methyl]naphthalene-1-ol **(4e)**

Yield = 86%; m.p. 224–226°C; Anal. Calcd. (%) for C_30_H_27_ClN_2_O: (Mol. Wt. = 466) C, 77.17; H, 5.78; N, 6.00. Found (%): C, 77.15; H, 5.80; N, 5.98. FT-IR (KBr, cm^−1^): 3127 br, *ν*(OH); 3044 *ν*(indole-NH); 751, *ν*(C–Cl). ^1^H NMR (*d*_6_-DMSO, ppm): 12.62 (s, 1H, phenolic OH, exchangeable with D_2_O); 9.95 (s, 1H, indole-NH); 7.31–7.81 (m, 14H, ArH); 4.99 (s, 1H, N–CH); 3.40 (t, 4H, 2x CH_2_); 2.50 (m, 2H, CH_2_); 1.13 (m, 4H, 2x CH_2_). ^13^C NMR (*d*_6_-DMSO, ppm): 186.00 (C–Cl), 150.51 (C–OH), 134.91, 130.60, 130.33, 129.80, 129.52, 127.47, 127.41, 124.20, 120.57, 114.16, 113.42, 40.50, 40.42, 40.33, 40.25, 40.16, 40.09, 40.00, 39.83, 39.66, 39.49. MS (EI) *m*/*z*: (M^+^, M^+2^): 466, 488 (5%, 1.5%).

#### 3.3.6. Preparation of 2-[(5-Methyl-2-phenyl-1*H*-indol-3-yl)(piperidin-1-yl)methyl]naphthalene-1-ol **(4f)**

Yield = 87%; m.p. 216–218°C; Anal. Calcd. (%) for C_31_H_30_N_2_O: (Mol. Wt. = 446) C, 83.37; H, 6.77; N, 6.27. Found (%): C, 83.41; H, 6.74; N, 6.24. FT-IR (KBr, cm^−1^): 3125 br, *ν*(OH); 3075 *ν*(indole-NH). ^1^H NMR (*d*_6_-DMSO, ppm): 12.49 (s, 1H, phenolic OH, exchangeable with D_2_O); 9.92 (s, 1H, indole-NH); 7.30–7.85 (m, 14H, ArH); 5.02 (s, 1H, N–CH); 3.39 (t, 4H, 2x CH_2_); 2.44 (m, 2H, CH_2_); 2.35 (s, 3H, C–CH_3_); 1.19 (m, 4H, 2x CH_2_).

#### 3.3.7. Preparation of 1-[(Diethylamino)(2-phenyl-1*H*-indol-3-yl)methyl]naphthalene-2-ol **(4g)**

Yield = 85%; m.p. 281–283°C; Anal. Calcd. (%) for C_29_H_28_N_2_O: (Mol. Wt. = 420) C, 82.85; H, 6.66; N, 6.66. Found (%): C, 82.82; H, 6.40; N, 6.56. FT-IR (KBr, cm^−1^): 3137 br, *ν*(OH); 3051 *ν*(indole-NH). ^1^H NMR (*d*_6_-DMSO, ppm): 12.51 (s, 1H, phenolic OH, exchangeable with D_2_O); 9.92 (s, 1H, indole-NH); 7.32–7.78 (m, 15H, ArH); 5.01 (s, 1H, N–CH); 2.59 (q, 4H, 2x CH_2_); 1.20 (t, 6H, 2x CH_3_).

#### 3.3.8. Preparation of 1-[(5-Chloro-2-phenyl-1*H*-indol-3-yl)(diethylamino)methyl]naphthalene-1-ol **(4h)**

Yield = 89%; m.p. > 300°C; Anal. Calcd. (%) for C_29_H_27_ClN_2_O: (Mol. Wt. = 454) C, 76.55; H, 5.98; N, 6.16. Found (%): C, 76.56; H, 5.94; N, 6.15. FT-IR (KBr, cm^−1^): 3126 br, *ν*(OH); 3045 *ν*(indole-NH); 750, *ν*(C–Cl). ^1^H NMR (*d*_6_-DMSO, ppm): 12.60 (s, 1H, phenolic OH, exchangeable with D_2_O); 9.96 (s, 1H, indole-NH); 7.30–7.81 (m, 14H, ArH); 4.97 (s, 1H, N–CH); 2.45 (q, 4H, 2x CH_2_); 1.25 (t, 6H, 2x CH_3_). ^13^C NMR (*d*_6_-DMSO, ppm): 185.99 (C–Cl), 150.50 (C–OH), 134.91, 130.58, 130.33, 129.81, 129.51, 127.47, 127.41, 124.19, 120.58, 114.14, 113.44, 40.50, 40.42, 40.33, 40.25, 40.16, 40.09, 39.99, 39.83, 39.66, 39.49. MS (EI) *m*/*z*: (M^+^, M^+2^): 454, 456 (4.5%, 1.1%).

#### 3.3.9. Preparation of 1-[(Diethylamino)(5-methyl-2-phenyl-1*H*-indol-3-yl)methyl]naphthalene-2-ol **(4i)**

Yield = 89%; m.p. 272–274°C; Anal. Calcd. (%) for C_30_H_30_N_2_O: (Mol. Wt. = 434) C, 82.91; H, 6.96; N, 6.45. Found (%): C, 82.94; H, 6.91; N, 6.44. FT-IR (KBr, cm^−1^): 3119 br, *ν*(OH); 3051 *ν*(indole-NH). ^1^H NMR (*d*_6_-DMSO, ppm): 12.51 (s, 1H, phenolic OH, exchangeable with D_2_O); 9.92 (s, 1H, indole-NH); 7.32–7.78 (m, 15H, ArH); 5.01 (s, 1H, N–CH); 2.59 (q, 4H, 2x CH_2_); 1.20 (t, 6H, 2x CH_3_).

#### 3.3.10. Preparation of 2-[(Diethylamino)(2-phenyl-1*H*-indol-3-yl)methyl]naphthalene-1-ol **(4j)**

Yield = 78%; m.p. 248–250°C; Anal. Calcd. (%) for C_29_H_28_N_2_O: (Mol. Wt. = 420) C, 82.82; H, 6.66; N, 6.66. Found (%): C, 82.84; H, 6.67; N, 6.65. FT-IR (KBr, cm^−1^): 3121 br, *ν*(OH); 3049 *ν*(indole-NH). ^1^H NMR (*d*_6_-DMSO, ppm): 12.49 (s, 1H, phenolic OH, exchangeable with D_2_O); 9.97 (s, 1H, indole-NH); 7.30–7.79 (m, 15H, ArH); 5.05 (s, 1H, N–CH); 2.48 (q, 4H, 2x CH_2_); 1.19 (t, 6H, 2x CH_3_).

#### 3.3.11. Preparation of 2-[(5-Chloro-2-phenyl-1*H*-indol-3-yl)(diethylamino)methyl]naphthalene-2-ol **(4k)**

Yield = 82%; m.p. 288–290°C; Anal. Calcd. (%) for C_29_H_27_ClN_2_O: (Mol. Wt. = 454) C, 76.55; H, 5.98; N, 6.16. Found (%): C, 76.57; H, 5.95; N, 6.16. FT-IR (KBr, cm^−1^): 3125 br, *ν*(OH); 3044 *ν*(indole-NH); 752, *ν*(C–Cl). ^1^H NMR (*d*_6_-DMSO, ppm): 12.59 (s, 1H, phenolic OH, exchangeable with D_2_O); 9.95 (s, 1H, indole-NH); 7.31–7.81 (m, 14H, ArH); 5.00 (s, 1H, N–CH); 2.41 (q, 4H, 2x CH_2_); 1.15 (t, 6H, 2x CH_3_). ^13^C NMR (*d*_6_-DMSO, ppm): 186.00 (C–Cl), 150.51 (C–OH), 134.91, 130.59, 130.33, 129.81, 129.51, 127.47, 127.42, 124.20, 120.57, 114.15, 113.43, 40.50, 40.43, 40.33, 40.26, 40.17, 40.09, 39.99, 39.83, 39.67, 39.50. MS (EI) *m*/*z*: (M^+^, M^+2^): 454, 456 (5%, 1.4%).

#### 3.3.12. Preparation of 1-[(Diethylamino)(5-methyl-2-phenyl-1*H*-indol-3-yl)methyl]naphthalene-2-ol **(4l)**

Yield = 84%; m.p. 232–234°C; Anal. Calcd. (%) for C_30_H_30_N_2_O: (Mol. Wt. = 434) C, 82.91; H, 6.96; N, 6.45. Found (%): C, 82.95; H, 6.92; N, 6.46. FT-IR (KBr, cm^−1^): 3120 br, *ν*(OH); 3040 *ν*(indole-NH). ^1^H NMR (*d*_6_-DMSO, ppm): 12.50 (s, 1H, phenolic OH, exchangeable with D_2_O); 9.93 (s, 1H, indole-NH); 7.30–7.77 (m, 14H, ArH); 5.03 (s, 1H, N–CH); 2.49 (q, 4H, 2x CH_2_); 1.19 (t, 6H, 2x CH_3_).

#### 3.3.13. Preparation of 1-[(Dimethylamino)(2-phenyl-1*H*-indol-3-yl)methyl]naphthalene-2-ol **(4m)**

Yield = 89%; m.p. 256–258°C; Anal. Calcd. (%) for C_27_H_24_N_2_O: (Mol. Wt. = 392) C, 82.62; H, 6.16; N, 7.14. Found (%): C, 82.65; H, 6.12; N, 7.15. FT-IR (KBr, cm^−1^): 3133 br, *ν*(OH); 3051 *ν*(indole-NH). ^1^H NMR (*d*_6_-DMSO, ppm): 12.45 (s, 1H, phenolic OH, exchangeable with D_2_O); 9.90 (s, 1H, indole-NH); 7.34–8.00 (m, 15H, ArH); 5.05 (s, 1H, N–CH); 2.25 (s, 6H, 2x CH_3_).

#### 3.3.14. Preparation of 1-[(5-Chloro-2-phenyl-1*H*-indol-3-yl)(dimethylamino)methyl]naphthalene-2-ol **(4n)**

Yield = 89%; m.p. 265–267°C; Anal. Calcd. (%) for C_27_H_28_ClN_2_O: (Mol. Wt. = 426) C, 75.96; H, 5.46; N, 6.56. Found (%): C, 75.95; H, 5.39; N, 6.55. FT-IR (KBr, cm^−1^): 3160 br, *ν*(OH); 3045 *ν*(indole-NH); 751, *ν*(C–Cl). ^1^H NMR (*d*_6_-DMSO, ppm): 12.60 (s, 1H, phenolic OH, exchangeable with D_2_O); 9.96 (s, 1H, indole-NH); 7.29–7.76 (m, 14H, ArH); 5.12 (s, 1H, N–CH); 2.38 (s, 6H, 2x CH_3_). ^13^C NMR (*d*_6_-DMSO, ppm): 185.99 (C–Cl), 150.50 (C–OH), 134.92, 130.58, 130.29, 129.82, 129.51, 127.48, 127.41, 124.18, 120.58, 114.14, 113.44, 40.49, 40.33, 40.25, 40.16, 39.9, 39.83, 39.66, 39.49. MS (EI) *m*/*z*: (M^+^, M^+2^): 454, 456 (4%, 1%).

#### 3.3.15. Preparation of 1-[(Dimetylamino)(5-methyl-2-phenyl-1*H*-indol-3-yl)methyl]naphthalene-2-ol **(4o)**

Yield = 84%; m.p. 281–283°C; Anal. Calcd. (%) for C_28_H_26_N_2_O: (Mol. Wt. = 406) C, 82.73; H, 6.45; N, 6.89. Found (%): C, 82.75; H, 6.40; N, 6.86. FT-IR (KBr, cm^−1^): 3137 br, *ν*(OH); 3051 *ν*(indole-NH). ^1^H NMR (*d*_6_-DMSO, ppm): 12.55 (s, 1H, phenolic OH, exchangeable with D_2_O); 9.98 (s, 1H, indole-NH); 7.32–7.79 (m, 14H, ArH); 5.09 (s, 1H, N–CH); 2.49 (s, 3H, CH_3_); 1.91 (s, 6H, 2x CH_3_).

#### 3.3.16. Preparation of 2-[(Dimethylamino)(2-phenyl-1*H*-indol-3-yl)methyl]naphthalene-1-ol **(4p)**

Yield = 88%; m.p. 228–230°C; Anal. Calcd. (%) for C_27_H_24_N_2_O: (Mol. Wt. = 392) C, 82.62; H, 6.16; N, 7.14. Found (%): C, 82.64; H, 6.13; N, 7.14. FT-IR (KBr, cm^−1^): 3140 br, *ν*(OH); 3041 *ν*(indole-NH). ^1^H NMR (*d*_6_-DMSO, ppm): 12.40 (s, 1H, phenolic OH, exchangeable with D_2_O); 9.91 (s, 1H, indole-NH); 7.33–7.99 (m, 15H, ArH); 5.02 (s, 1H, N–CH); 2.20 (s, 6H, 2x CH_3_).

#### 3.3.17. Preparation of 2-[(5-Chloro-2-phenyl-1*H*-indol-3-yl)(dimethylamino)methyl]naphthalene-1-ol **(4q)**

Yield = 84%; m.p. 240–242°C; Anal. Calcd. (%) for C_27_H_28_ClN_2_O: (Mol. Wt. = 426) C, 75.96; H, 5.46; N, 6.56. Found (%): C, 75.93; H, 5.39; N, 6.55. FT-IR (KBr, cm^−1^): 3125 br, *ν*(OH); 3043 *ν*(indole-NH); 751, *ν*(C–Cl). ^1^H NMR (*d*_6_-DMSO, ppm): 12.59 (s, 1H, phenolic OH, exchangeable with D_2_O); 9.95 (s, 1H, indole-NH); 7.30–7.81 (m, 14H, ArH); 5.10 (s, 1H, N–CH); 2.19 (s, 6H, 2x CH_3_). ^13^C NMR (*d*_6_-DMSO, ppm) 185.99 (C–Cl), 150.50 (C–OH), 134.92, 130.58, 130.33, 129.81, 129.51, 127.48, 127.41, 124.18, 120.58, 114.14, 113.44, 40.49, 40.33, 40.25, 40.16, 40.09, 39.98, 39.83, 39.66, 39.49. MS (EI) *m*/*z*: (M^+^, M^+2^):: 454, 456 (5%, 1.6%).

#### 3.3.18. Preparation of 2-[(Dimetylamino)(5-methyl-2-phenyl-1*H*-indol-3-yl)methyl]naphthalene-1-ol **(4r)**

Yield = 82%; m.p. 254–256°C; Anal. Calcd. (%) for C_28_H_26_N_2_O: (Mol. Wt. = 406) C, 82.73; H, 6.46; N, 6.89. Found (%): C, 82.75; H, 6.40; N, 6.88. FT-IR (KBr, cm^−1^): 3120 br, *ν*(OH); 3039 *ν*(indole-NH). ^1^H NMR (*d*_6_-DMSO, ppm): 12.49 (s, 1H, phenolic OH, exchangeable with D_2_O); 9.97 (s, 1H, indole-NH); 7.30–7.78 (m, 14H, ArH); 5.04 (s, 1H, N–CH); 2.39 (s, 3H, CH_3_); 1.93 (s, 6H, 2x CH_3_).

## 4. Conclusions

In the conclusion, the present study revealed that the compounds having methyl and chlorosubstituent exhibited good antioxidant, antimicrobial, and cytotoxic activity.

## Figures and Tables

**Table 1:** Synthesis of [(disubstituted amino)(5-substituted 2-phenyl-1*H*-indol-3-yl)methyl]naphthalene-ols (**4a–4r**).

Starting materials				Products (**4a–4r**)	Time (h)	Yield (%) isolated
Entry	Amines	R	Naphthol			
1	Piperidine	H	2-Naphthol	**4a**	3	82
2	Piperidine	Cl	2-Naphthol	**4b**	3	85
3	Piperidine	CH_3_	2-Naphthol	**4c**	2	79
4	Piperidine	H	1-Naphthol	**4d**	2	89
5	Piperidine	Cl	1-Naphthol	**4e**	3	86
6	Piperidine	CH_3_	1-Naphthol	**4f**	3	87
7	Diethyl amine	H	2-Naphthol	**4g**	3	90
8	Diethyl amine	Cl	2-Naphthol	**4h**	2	89
9	Diethyl amine	CH_3_	2-Naphthol	**4i**	3	89
10	Diethyl amine	H	1-Naphthol	**4j**	3	78
11	Diethyl amine	Cl	1-Naphthol	**4k**	3	82
12	Diethyl amine	CH_3_	1-Naphthol	**4l**	2	84
13	Dimethyl amine	H	2-Naphthol	**4m**	3	89
14	Dimethyl amine	Cl	2-Naphthol	**4n**	3	89
15	Dimethyl amine	CH_3_	2-Naphthol	**4o**	3	85
16	Dimethyl amine	H	1-Naphthol	**4p**	3	88
17	Dimethyl amine	Cl	1-Naphthol	**4q**	3	84
18	Dimethyl amine	CH_3_	1-Naphthol	**4r**	2	82

**Table 2:** RSA of the test compounds (**4a–4r**) and standards using DPPH scavenging method.

Compounds	Concentration				
	25 *μ*g/mL (%)	50 *μ*g/mL (%)	75 *μ*g/mL (%)	100 *μ*g/mL (%)	IC_50_ (*μ*g/mL)
**4a**	68.81	70.73	70.41	70.41	<25
**4b**	72.05	72.66	71.70	75.88	<25
**4c**	42.12	40.19	42.44	44.05	<25
**4d**	50.16	57.23	58.84	61.09	<25
**4e**	78.77	84.56	86.17	85.53	<25
**4f**	71.06	72.99	68.48	71.06	<25
**4g**	69.13	69.45	72.34	68.16	<25
**4h**	67.84	71.70	74.27	77.49	<25
**4i**	68.16	69.13	70.09	69.13	<25
**4j**	65.91	68.16	71.70	70.09	<25
**4k**	81.02	80.38	79.09	77.81	<25
**4l**	65.91	71.06	69.13	69.77	<25
**4m**	44.37	37.29	44.05	44.69	<25
**4n**	85.20	77.81	78.45	78.13	<25
**4o**	70.09	75.24	74.59	74.59	<25
**4p**	70.09	72.99	73.63	73.64	<25
**4q**	78.45	81.67	83.27	78.77	<25
**4r**	78.45	81.67	83.27	78.77	<25
BHA	94.21	93.56	93.89	94.53	<25
TBHQ	93.24	91.96	94.21	94.85	<25
**AA**	91.63	92.92	93.24	93.89	<25

**Table 3:** *In vitro* antimicrobial activities of compounds (**4a–4r**).

Compound code	Antibacterial activity (MIC *μ*g/mL)				Antifungal activity (MIC *μ*g/mL)			
	EC^a^	SA^b^	KP^c^	PA^d^	AO^e^	AN^f^	AF^g^	AT^h^
**4a**	512	1024	512	1024	512	256	256	256
**4b**	64	32	64	128	128	128	32	64
**4c**	128	128	256	128	256	128	128	256
**4d**	256	512	1024	512	1024	512	256	256
**4e**	64	64	128	64	64	128	64	128
**4f**	128	512	256	256	128	256	128	256
**4g**	1024	256	512	512	256	256	256	256
**4h**	16	16	64	64	16	16	64	64
**4i**	256	128	256	256	128	256	128	256
**4j**	512	1024	512	512	256	512	512	512
**4k**	16	16	32	32	16	16	64	32
**4l**	256	256	128	128	256	256	256	256
**4m**	512	512	512	1024	512	256	256	512
**4n**	16	16	08	16	08	08	16	08
**4o**	128	128	64	128	128	64	128	128
**4p**	256	256	256	512	128	128	256	256
**4q**	16	08	16	08	32	08	16	16
**4r**	64	32	64	32	32	32	32	32
Gentamycin	02	02	02	02	—	—	—	—
Fluconazole	—	—	—	—	02	02	02	02

^a^EC: *Escherichia coli* (MTCC-723), ^b^SA: *Staphylococcus aureus* (ATCC-29513), ^c^KP: *Klebsiella pneumonia* (NCTC-13368), ^d^PA: *Pseudomonas aeruginosa* (MTCC-1688), ^e^AO: *Aspergillus oryzae* (MTCC-3567^T^), ^f^AN: *Aspergillus niger* (MTCC-281), ^g^AF: *Aspergillus flavus* (MTCC-1973), and ^h^AT: *Aspergillus terreus* (MTCC-1782).

**Table 4:** Antitubercular activity of compounds (**4a–4r**) against *Mycobacterium tuberculosis H37Rv*.

Compound number	MIC^a^ values (*μ*g/mL)
**4a**	50
**4b**	25
**4c**	50
**4d**	50
**4e**	25
**4f**	25
**4g**	50
**4h**	12.5
**4i**	25
**4j**	25
**4k**	12.5
**4l**	25
**4m**	25
**4n**	12.5
**4o**	25
**4p**	50
**4q**	6.25
**4r**	12.5
Pyrazinamide	3.125
Streptomycin	6.25

**Table 5:** Anticancer activity of compounds** (4a–4r)**.

Compound	Concentration (*μ*g/mL)	OD at 492 nm	% of cell lysis	IC_50_ (*μ*g/mL)
**4b**	10	0.799	75%	<10 *μ*G
**4b**	20	0.885	>75%	
**4b**	30	1.559	100%	
**4e**	10	0.701	>50%	<10 *μ*G
**4e**	20	0.915	75%	
**4e**	30	1.010	>75%	
**4h**	10	0.453	<50%	20 *μ*G
**4h**	20	0.550	50%	
**4h**	30	0.799	75%	
**4k**	10	1.333	100%	Very <10 *μ*G
**4k**	20	1.548	100%	
**4k**	30	1.873	100%	
**4n**	10	1.143	>75%	<10 *μ*G
**4n**	20	1.734	100%	
**4n**	30	1.822	100%	
**4q**	10	0.956	100%	Very <10 *μ*G
**4q**	20	1.392	100%	
**4q**	30	1.752	100%	
**4r**	10	0.377	No lysis	20 *μ*G
**4r**	20	0.606	50%	
**4r**	30	0.700	>50%	
Control	—	0.349	No lysis	

Cell line-**MDA-MB-**human adenocarcinoma, mammary gland.
